# Ameliorative Effects of Different Transcranial Electrical Stimulation Paradigms on the Novel Object Recognition Task in a Rat Model of Alzheimer Disease

**DOI:** 10.31661/gmj.v8i0.1440

**Published:** 2019-03-30

**Authors:** Amir Hossein Zarifkar, Asadollah Zarifkar, Mohammad Nami, Ali Rafati, Hadi Aligholi, Farzaneh Vafaee

**Affiliations:** ^1^Department of Physiology, School of Medicine, Shiraz University of Medical Sciences, Shiraz, Iran; ^2^Department of Neuroscience, School of Advanced Medical Sciences and Technologies, Shiraz University of Medical Sciences, Shiraz, Iran; ^3^DANA Brain Health Institute, Iranian Neuroscience Society, Fars Chapter, Shiraz, Iran

**Keywords:** Alzheimer Disease, Memory, Cognitive Impairment, Novel Object Recognition Test, tES

## Abstract

**Background:**

Treatment of Alzheimer as a disease that is associated with cognitive impairment has been associated with some restrictions. Recently, researchers have focused on non-pharmacological treatments, including non-invasive stimulation of the brain by transcranial electrical stimulation (tES). Four main paradigms of transcranial electrical current include transcranial direct current stimulation (tDCS), transcranial alternative current stimulation (tACS), transcranial random noise stimulation (tRNS), transcranial pulse current stimulation (tPCS). The tDCS is a possible new therapeutic option for patients with cognitive impairment, including Alzheimer disease.

**Materials and Methods:**

The study was done on Sprague-Dawley male rats weighing 250-270 g. to develop Alzheimer’s model, the cannula was implanted bilaterally into the hippocampus. Aβ 25-35 (5μg/ 2.5µl/day) was microinjected bilaterally for 4 days. Then, an electrical stimulation paradigm was applied to the animal for 6 days. Animal cognitive capacity was evaluated on day 11 and 12 by novel object recognition (NOR) test.

**Results:**

Our results showed that application of tDCS; tACS; tRNS and tPCS reversed beta-amyloid-induced impairment (P<0.05). The tRNS Group spent total exploration time around the objects compared to other groups (P<0.05). There was no significant difference between the four different paradigms in discrimination ratio and the percentage of total exploration time.

**Conclusion:**

The results of this study showed that the use of multiple sessions of different tES paradigms could improve Aβ-induced memory impairment in the NOR test. Therefore, based on evidence, it can be expected that in addition to using tDCS, other stimulatory paradigms may also be considered in the treatment of AD.

## Introduction

Alzheimer disease (AD) is a progressive and irreversible neurodegenerative disorder which eventually leads to amnesia. AD affects cognitive and behavioral functions as a result of synaptic dysfunction. It is associated with cognitive decline, neurotoxicity, and the formation of extracellular plaques, mainly of beta-amyloid (Aβ) peptides and intercellular neurofibrillary tangles, consisting of the hyperphosphorylation tau protein [1-4]. Previous studies have shown that Aβ injection causes pathological effects on learning and memory processes. This impairment is caused not only by Aβ 1-40 and Aβ 1-42 but also caused by the C-terminal fragment of the molecule, namely Aβ 25-35 [5, 6]. The effects created by this fragment are like a whole fragment [7]. In animal studies, intrahippocampal injection of Aβ 25-35 causes learning disruption and histological and biochemical changes. Therefore, these animals are used as one of the AD models [8, 9]. Regarding the therapeutic constraints of AD by medication; induction of neuroplastic changes by non-invasive transcranial electrical stimulation (tES) techniques has been increasing in recent years [10]. This technique is accomplished by generating direct current on the skull surface by using electrodes that are embedded in rubber coated with a sponge that is damped with saline or guiding gels [11]. This technique can make certain changes based on duration and polarity of the electrodes in the excitability of human motor cortex. In the most common method, an electrode is placed in a specific area, while another electrode is placed in another area to establish an electrical current [12]. The position of the electrodes is necessary to determine the orientation and spatial distribution of the current and ultimately the effectiveness of the treatment [13]. This method is a valuable tool in the treatment of many neuropsychiatric disorders such as depression, anxiety, chronic pain, Parkinson disease, and AD, as well as in the rehabilitation of cognitive processes [14]. Four main paradigms of tES include transcranial direct current stimulation (tDCS), transcranial alternative current stimulation (tACS), transcranial random noise stimulation (tRNS), transcranial pulse current stimulation (tPCS). The tDCS is a method to control the neuronal transmembrane potential by flowing a weak current to the scalp. The tDCS modulates spontaneous neuronal network activity through polarization of the resting membrane potential, rather than causing neuronal firing by suprathreshold neuronal membrane depolarization. The effect of tDCS depends on the direction of current polarity of the electrodes; anodal stimulation increases cortical activity and excitability, while cathodal stimulation decreases. The effects of tDCS are observed not only during stimulation but also after the end of stimulation (after-effect). The factors that affect stimulation are duration, intensity, the polarity of stimulation and baseline cortical excitability state [15, 16]. The tACS uses an electrical current that alternates between electrodes, in a sinusoidal wave. Unlike tDCS, tACS does not alter neuronal excitability but entrains the neuronal firing from a large number of underlying neurons to the exogenous frequency [17]. Neuronal entrainment is achieved by the applied current altering the transmembrane potential of neurons. The polarization of neurons reflects the current applied to it, leading to a sinusoidal fluctuation of the membrane potential. As this fluctuation is both frequencies dependent and linearly proportional to the applied current, lower-frequency stimulation induces larger polarization than does higher frequencies. Unlike tDCS, which has inhibitory effects due to polarity, the effects of tACS are determined by the current frequency and independent of the polarization of the electrodes [18]. The tRNS is a special form of tACS that involves the application of random noise oscillations above selected brain regions to modulate cortical plasticity. One of the proposed mechanisms of tRNS is the increase of neuronal excitability via stochastic resonance, whereby weak neural signal detection in the central nervous system is enhanced when noise is added. The advantages of this new technique, compared with tDCS, include the lack of sensitivity to the polarity of the electrodes and the reduction of skin sensitivity to the electrodes during stimulation [19].The tPCS is a direct current stimulation with a non-constant current in which the current is applied with a constant amplitude. In this paradigm, the stimulation is interrupted at regular intervals, and the definitions of pulse duration, frequency, and inter-pulse intervals are added to the current, and therefore a special stimulation form is created [20]. In previous studies, the effects of tPCS have been studied in some clinical conditions such as depression, anxiety and pain disorders [21-23]. It has also been shown that tPCS has potential benefits for cognitive functions [24]. Compared to the other three paradigms, tDCS has become more known and studied, and its mechanisms have been further investigated. Clinical studies have shown that tDCS is considered as a therapeutic tool. Many studies have shown that tDCS is used to treat many disorders, including those that do not respond to drug therapy, including post-stroke motor disorder [25], aphasia after stroke [26], epilepsy [27], chronic pain [28], and Parkinson disease [29]. Several studies have also shown that the use of tDCS can improve memory in AD [30, 31]. The tDCS can improve descriptive memory and working memory, as well as other cognitive functions not only in patients but in healthy people [32, 33]. The precise mechanism responsible for the effects of tDCS has not been fully described, so further studies are needed for its clinical application. It has been determined that the use of an electric field with sufficient strength and time will increase the electrical conductivity of biological membranes. This is due to increased permeability for small and large ions and molecules. However, knowledge about the effects of neurotransmitters, neurological markers, neural pathways, or neural interactions is incomplete. It has been shown that tDCS in AD causes changes in neuronal activity, blood flow to the brain, osmotic brain activity, communication patterns of the brain, synaptic and non-synaptic effects, and neural modulation. Therefore, due to the mechanisms of action and mechanisms involved in tDCS disease, it can be used as a suitable treatment to improve cognitive function in AD [34]. Several studies have been conducted using tDCS in AD [34]. However, the number of animal studies, which are using this technique to find out the mechanisms of this technique is increasing. Yu *et al*. showed that tDCS application after the onset of cognitive dysfunction caused by AD leads to a positive effect on motor behavior [35]. Ronso *et al.* in 2017 demonstrated that tDCS with training improves cognition in anomic AD and frontotemporal dementia [36]. In a case study; the use of tDCS as an adjuvant to the traditional treatment had a positive effect on overall patient cognitive function and improved performance on all the secondary outcome measures [37]. In another study shown the synergetic application of tDCS and cognitive training led to slow down the cognitive decline in AD [38]. Considering the impairing effects of Aβ on cognitive function and suggested neuroprotective effect of tES, this study was designed to comparatively evaluate the effects of different electrical stimulation paradigm on cognitive impairment induced by Aβ 25-35 in novel object recognition (NOR) test and finally to determine which of the tES paradigms are more effective in this regard.

## Materials and Methods

### Animals

Adult male Sprague-Dawley rats weighing 250–270g were used. Animals were maintained at room temperature (25 ± 2 °C) under standard 12–12h light-dark cycle with lights on at 7:00 A.M. Food and water were available *ad libitum*. The experimental protocols were approved by the ethics committee of Shiraz University of Medical Sciences (IR.SUMS.REC.1395.S974), and the animal care was according to the NIH Guide for the care and use of laboratory animals. Fifty-six rats were randomly divided into the seven groups (n=8 per each group); the control group (cage control), the sham group, the Aβ group, the Aβ + tDCS group, the Aβ + tACS group, the Aβ + tRNS group, and the Aβ + tPCS group.

### Materials and Reagents

Aβ 25-35 was purchased from Sigma (USA), and the electrical stimulation device was purchased from Medina Teb Company (Iran). Ketamine and xylazine were provided by Alfasan Woerden Company (Netherlands).

### Surgery

On the day of surgery, rats were anesthetized with intraperitoneal injection of mixed Ketamine (100mg/kg) and xylazine (10mg/kg). The rats were mounted into a stereotaxic frame (Stoelting Company, USA) and according to Paxinos brain atlas, stainless steel guide cannula (22-gauge) were implanted bilaterally into the dorsal hippocampus (AP−3.8, ML ± 2.2 DV−2.7). To apply electrical stimulation, a plastic tube (inner diameter: 2 mm) was mounted on the right frontal cortex. The cannula and plastic tube were anchored to the skull using stainless screws and acrylic cement.

### Aβ 25-35 Preparation

Aβ peptide (25-35) was dissolved in sterile distilled water at a concentration of 2 μg/μl and was stored in −70 °C. Aggregation of Aβ 25-35 was done by in-vitro incubation at 37 °C for 4 days [39].

### Drug Administration

In order to inject the drug, a 10 µl Hamilton syringe was connected to the injection cannula through a short piece of polyethylene tube; the injection cannula was inserted 0.5mm the tip of the guide cannula. Aβ 25-35 (5 μg/ 2.5 µl/day) or its vehicle (distilled water) was injected bilaterally in the four doses on days 1 and 4. All microinjections were carried out at the speed of 1 µl/min by microinjection pump, and the needle was left in the place for an additional 5min to minimize the back-flow of the solution.

### Induction of Electrical Stimulation

The plastic tube which was placed on the skull surface on surgery day was filled with sponge. Rats were covered with a towel, and the electrodes were inserted. The anodal electrode was placed into the plastic tube above the right frontal cortex. The cathodal electrode, with a larger contact area, was placed onto the ventral thorax with a corset. To reduce the contact impedance, sponges were moistened with saline solution prior to electrical stimulation. The tES was applied to the awake and freely moving rats for one week, 20 min per session, with current intensities of 200 μA, the current intensity was ramped for 10s. Sham stimulation, (electrodes were placed, but no stimulation was applied) was performed in the sham and the Aβ groups. Ten days after surgery (day 11), behavioral test (NOR test) was carried out.

### NOR Test

This test is made up of a test box with dimensions 65× 45 × 65 cm. The protocol consists of two days. On the first day, the rats are placed in the box for 5 min without any objects, to familiarize to the test box. On the second day (test day), the rats were placed in a test box, and two objects were placed in two corners (about 30 cm apart). Objects used in this study are plastic blocks in the same size, shape, and color. The time taken to check each object within 5 minutes (as defined training session) was recorded. The rats then returned to the cage. After a period of 60 min, the rats were re-tested in the test box, and at this stage, one of the familiar objects used in the previous training session was replaced with a new object. The time taken to check each object was recorded within 5 min (as defined test session). The animals were considered to be exploring when they were facing, sniffing or biting the object. The test box and objects were cleaned with 70% of ethanol between trials. A discrimination index (the time spent with the novel object divided by the total time spent exploring either object) was used to measure memory preference.

### Data Analysis

All behavioral tests and decoding were performed blind. All statistical tests were undertaken using SPSS v22.0 (SPSS Inc., Chicago, Ill., USA). Normality of data distribution was checked by using the Shapiro–Wilk test. Data were analyzed by one-way analysis of variance (ANOVA) followed by post hoc LSD test for multiple comparisons. Object exploration time converted to the percentage of total exploration time, and a one-sample t-test was used to compare the percentage of total time of exploration spent on each object considering a theoretical mean of 50%. All results have been shown as means ± Standard Error of Mean )S.E.M (. In all statistical comparisons, P<0.05 was considered as significant difference.

## Results

**Figure 1:**
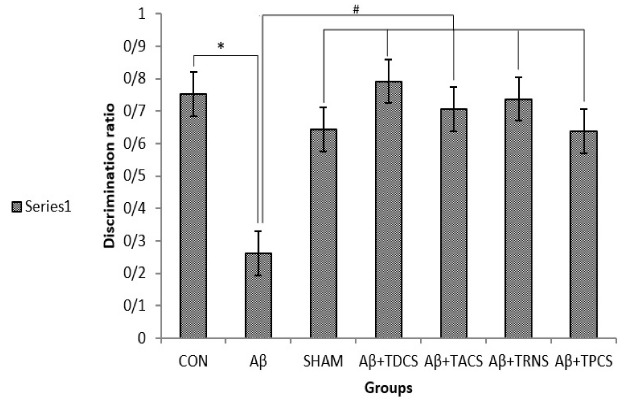
The effect of vehicle, Aβ 25-35 or/and tDCS; tACS; tRNS; tPCS on discrimination ratio. Discrimination ratio has significant difference between groups (P=0.003). *P < 0.01 and #P< 0.001 represents the difference between animals, which receive Aβ25-35, sham, and Aβ25-35 + tDCS; tACS; tRNS; tPCS (n=8 per group).

**Figure 2:**
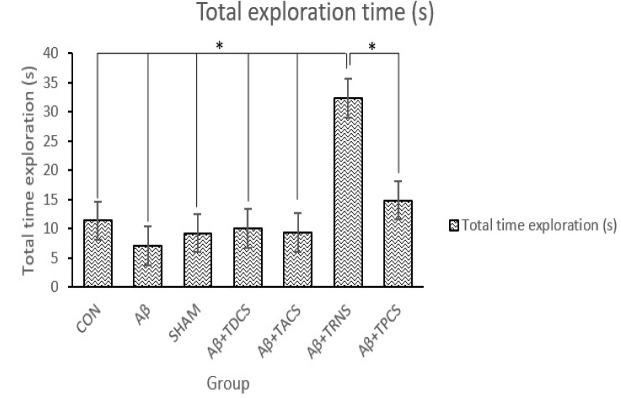
The effect of vehicle, Aβ 25-35 or/and tDCS; tACS; tRNS; tPCS on total exploration time around the objects (P = 0.02). *P<0.05 represents the difference between Aβ25-35+tRNS animals and animals, which recieve Aβ25-35, sham, and Aβ25-35 + tDCS; tACS; tPCS (n=8 per group).

**Figure 3:**
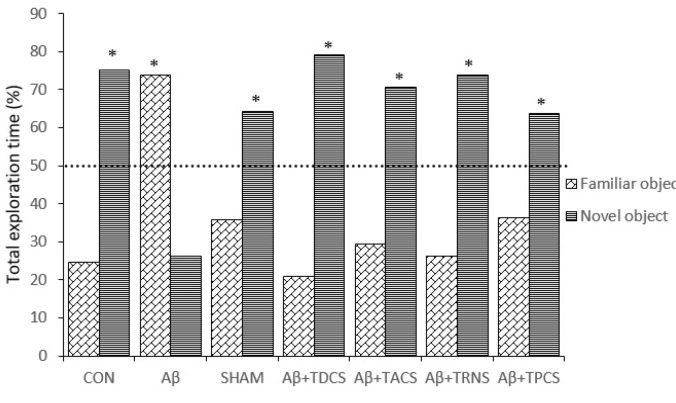
The effect of vehicle, Aβ 25-35 or/and tDCS; tACS; tRNS; tPCS on the percentage of total exploration time around the objects. Data are shown as mean and S.E.M of the percentage of total exploration time. *P<0.05 on one sample t-test, n = 8 per group)

The effects of the vehicle; Aβ or/and tDCS; tACS; tRNS; tPCS on NOR test is represented in Figure-1 and 2. Figure-1 shows the discrimination ratio (the time spent with the novel object divided by the total time spent exploring either object) between groups. ANOVA analysis showed a significant difference in discrimination ratio between groups (P=0.003, F [8, 55] = 4.133). Post hoc LSD test following ANOVA analysis revealed that discrimination ratio in Aβ receiving group is significantly decreased compared with the other groups. Aβ group rats showed deficits in NOR. Figure-2 demonstrates only the group who received tRNS had a significant difference with the rest of the groups and spent more time around the objects (P= 0.02, F[8,55]=2.886). In terms of the percentage of total exploration time around each object; Aβ group has spent less time than other groups around the novel object (mean; novel object = 26.19%; familiar object = 73.80%, P> 0.05; Figure-3). Animals in control, sham and Aβ25-35 + tDCS; tACS; tRNS; tPCS groups explored novel object for a greater percentage of total exploration time around novel object (mean of these groups, control; novel object=75.28%; familiar object=24.71%; P<0.05, sham; novel object = 64.28%; familiar object=35.71%; P<0.05, Aβ25-35 + tDCS; novel object=79.18%; familiar object=20.81%; P<0.05, Aβ25-35 + tACS; novel object =70.54%; familiar object =29.45%; P<0.05, Aβ25-35 + tRNS; novel object=73.71%; familiar object=26.28%; P<0.05, Aβ25-35 + tPCS; novel object=63.70%; familiar object=36.29%; P<0.05; Figure-3). These groups did not show deficits in NOR (P=0.05). Application of tDCS; tACS; tRNS and tPCS reversed Aβ-induced impairment. In these groups, there was a significant difference in the percentage of total exploration time around the novel object and familiar object (P<0.05).

## Discussion

The findings of this study revealed that repeated administration of Aβ 25-35 induced cognitive impairment in NOR test. It has been shown that this method could be a more reliable way to induce a consistent and less variable model of AD in rats [40]. The findings of the present study revealed thatdifferent paradigms of tES prevented Aβ-induced cognitive impairment in the NOR test. Previous studies showed that tDCS affected the brain cortex below the stimulation electrode, the path of current flowing between the electrodes penetrates not only the cortex but also sub-cortical structures including the hippocampus [41]. The tDCS affected the brain cortex below the stimulation electrode, the neurophysiological, behavioral and molecular changes investigated in the previous study were related to hippocampal function. Indeed, anodal tDCS enhanced long-term potentiation at hippocampal CA3-CA1 synapses and improved spatial and recognition memory assessed by two validated behavioral tests of hippocampal-dependent memory, i.e., Morris water maze and NOR [42]. The result of this study confirmed the results of the previous studies, with the difference that in this study, six sessions of electrical stimulation were carried out and also, these four paradigms also had amelioration effects on animal behavior. Forasmuch as, setting up the water maze is a complicated procedure and the testing condition is somewhat stressful to the animals. A more simple and friendly behavioral test would be helpful to evaluate a large number of potentially beneficial compounds in AD animal models. The previous study demonstrated that the NOR test is a facile and sensitive behavioral test in APP/PS1 AD model [43]. The NOR test is based on the spontaneous behavior of rodents to explore novelty and is a pure working memory test free of reference [44]. Hippocampus is important in the formation of recognition memory [45]. The two advantages of NOR test compared to other behavioral tests are that, firstly, this test is relatively simple and friendly. This test does not require spatial learning and the use of positive or negative reinforcement stimuli. A major problem in testing a water maze or shuttle box is the involvement of negative stimuli, such as deep water or electric shock. These stimuli may cause stress or even depression in rodents. Stress as a negative factor affects learning and memory [46, 47]. The features of this test are comparable to those commonly used in human memory tests. Secondly, this test requires a shorter period and is more repeatable. The simplicity of this test allows a large number of animals to be evaluated in a short time. In the previous study, it has been shown that anodal tPCS with a specific pulse duration has significant effects on corticospinal excitability compared to tDCS in healthy people [20]. However, in this study, all four methods had similar effects, and no significant difference was observed between them.In our study, the effect of different paradigms of tES on Aβ 25-35 induced cognitive impairment in the NOR test was investigated. Aβ efficiently disrupted the recognition memory for the NOR test; a task used to evaluate recognition memory performance in rodents [43, 48]. This issue was confirmed in the present study, and the effect of four paradigms of transcranial electrical stimulation on this disorder was investigated.

The groups that received tDCS, tACS, tRNS and tPCS stimulation took significant time around the novel object compared to Aβ groups. The Aβ group spent more time around the familiar object than the novel object. Other groups spent more time around the novel object. In the previous studies, it has been shown that anodal tDCS enhances long term potentiation in the mouse hippocampus and improves memory and spatial learning [49]. In this study, all four electrical stimulation paradigms improved memory impairment induced by Aβ in the NOR test. Unlike tDCS the other three stimulation patterns, it has not been studied much, and in this study, we examined the effect of three other stimuli on cognitive function. Past studies have shown that tACS can modulate cortical excitability and EEG oscillations and cognitive processes [50-52]. Also, it has been demonstrated that tACS can modulate brain oscillations and affect cognitive functions such as memory due to the relationship between brain oscillations and cognitive processes [53, 54]. In the present study, the effect of this paradigm on cognition was determined. It has also been shown that these functions change in brain oscillations with selective intervention [55]. Abnormal brain rhythms are associated with pathologic conditions. As shown in a study, these rhythms vary in Alzheimer patients [56]. Thus, the researchers are trying to the treatment of these neurological diseases by modulating these brain rhythms, and tACS paradigm with the application of a specific frequency creates this ability. The prevailing hypothesis about the action of tACS is that alternating fields can increase or decrease the power of oscillatory rhythms in the brain, and in the frequency dependent manner, through synchronizing and desynchronizing neuronal networks [18]. This study could partly prove the positive effects of tACS in this regard. Previous studies have shown that transcranial high-frequency random noise stimulation increases the brain excitability [57, 58]. In a study by Mulquiney *et al*., it has been shown that tRNS can improve working memory performance [59]. In a comparison of tDCS, tACS, and tRNS, one study in 2016 showed that tRNS is the most effective tES method for increasing cortical excitability of the motor cortex [60]. In our study, the effect of tRNS on the improvement of the performance of memory-impaired rats in NOR test was shown and had significant differences in total exploration time compared to other groups, and in this case, it seems more effective than other paradigms. In the previous study, it has been shown that anodal tPCS with a specific pulse duration has significant effects on corticospinal excitability compared to tDCS in healthy people [20]. In our study, tPCS did not significant difference compared to the other paradigm but could improve the Aβ-induced deficit in NOR test. However, regarding the number of stimulation sessions, the results of our study showed that all four paradigms had significant effects on the NOR test. According to the results of our study and previous studies, the effect of tDCS on the improvement of memory impairment induced by Aβ in NOR test seems to be well supported. Besides, current research has suggested that other stimulation paradigms may retain the efficiency in remediating cognitive impairment in an AD rodent model. Overall, the results of this study showed that the use of multiple sessions of different paradigms of tES could improve the memory impairment induced by Aβ in a rat model. Therefore, based on such evidence, it could be expected that, in addition to the use of tDCS in the treatment of AD, other stimulatory paradigms may also be considered as treatments in AD. However, more research is needed to make these methods available in clinical settings.

## Acknowledgment

This work was derived from the Ph.D. thesis of Amir Hossein Zarifkar and supported by a grant number:12527 from Shiraz University of Medical Sciences.

## Conflict of Interest

Authors have no conflict of interests.
